# Dihydroartemisinin–Piperaquine Combination in the Treatment of Uncomplicated *Plasmodium falciparum* Malaria: Update on Clinical Failures in Africa and Tools for Surveillance

**DOI:** 10.3390/jcm13226828

**Published:** 2024-11-13

**Authors:** Océane Delandre, Bruno Pradines, Emilie Javelle

**Affiliations:** 1 Unité Parasitologie et Entomologie, Département Microbiologie et Maladies Infectieuses, Institut de Recherche Biomédicale des Armées, 13005 Marseille, France; bruno.pradines@gmail.com (B.P.); emilie.javelle@gmail.com (E.J.); 2 Aix Marseille Univ, SSA, AP-HM, RITMES, 13005 Marseille, France; 3 IHU Méditerranée Infection, 13005 Marseille, France; 4 Centre National de Référence du Paludisme, 13005 Marseille, France

**Keywords:** malaria, *Plasmodium falciparum*, dihydroartemisinin–piperaquine, Eurartesim, *Pfk13*, *Pfpm2*, *Pfcrt*, molecular marker, resistance, RSA-PSA, in vitro

## Abstract

Dihydroartemisinin (or artenimol)–piperaquine is one of the six artemisinin-based combination therapies recommended in uncomplicated malaria treatment. However, artemisinin partial resistance has been reported in Cambodia, Laos, Vietnam, India, and, recently, in Africa. Polymorphisms in the *Pfk13* gene have been described as molecular markers of artemisinin resistance and the amplification of the plasmepsine II/III (*Pfpmp2*/*Pfpmp3*) gene has been associated with piperaquine resistance. However, some therapeutic failures with this combination remain unexplained by strains’ characterization. We provide an overview on the use of dihydroartemisinin–piperaquine in malaria treatment and discuss tools available to monitor its efficacy.

## 1. Background

Malaria disease is caused by an intraerythrocytic protozoan of the *Plasmodium* genus, and six plasmodial species are listed as infecting humans: *Plasmodium vivax*, *Plasmodium ovale curtisi*, *Plasmodium ovale wallikeri*, *Plasmodium malariae*, *Plasmodium knowlesi*, and *Plasmodium falciparum*. In the last world malaria report, published in 2023, the World Health Organization (WHO) estimated that 249 million cases of malaria occurred worldwide in 2022, compared to 244 million cases in 2020 [1]. This disease remains endemic in 84 countries, with 29 countries accounting for 96% of the global cases and the African region accounting for around 95% of the worldwide cases. Children under five years old are the most affected by the disease and represented 76% of the mortality due to malaria in 2015 and 80% in 2020. Almost half of the world’s population is exposed. Malaria is not only a burden in tropical areas but also in Europe where 4856 malaria imported cases were declared in 2021 [2]. In 2023, 3012 cases of imported malaria into mainland France were reported to the malaria French national center (Centre national de reference du paludisme) (personnal data). Malaria severity may differ according to the infecting plasmodial species. Underlying mechanisms of malaria complications include deformation, aggregation and rupture of infected red blood cells, obstruction of the micro-vessels, and system immune activation [3]. The uncomplicated plasmodial infection is characterized by unspecific signs such as fever, chills, headaches, myalgia, digestive disorders, splenomegaly, or hepatomegaly, without any WHO criteria for severity [4].

Morphologically, *Plasmodium* is composed of a nucleus, a mitochondrion, an apicoplast, a digestive vacuole, and other organelles common to apicomplexes. The mitochondrion and apicoplast are two organelles with their own circular genomes of 6 kb and 35 kb, respectively. The apicoplast is essential to the parasite’s survival and hosts various metabolic pathways such as fatty acid synthesis [5]. The digestive vacuole is a unique function dedicated to hemoglobin consumption and heme detoxification [6]. Due to its membrane transporters, it enables the efflux of compounds toxic to the parasite, making it a valuable drug target [6].

There are rising concerns about *P. falciparum* resistance to antimalarial drugs, which is not a recent phenomenon as it was described in 1910 towards quinine [7]. The massive production of chloroquine during the 1950s gave wide access to a cheap, safe, and effective malaria treatment [7,8], but consequently, clinical isolates harboring chloroquine resistance were reported as early as 1957–1960 in Colombia, Venezuela, Cambodia, Thailand, Papua New Guinea, and the Philippines [8]. Since then, resistances have emerged against each of the new antimalarial drugs that have been licensed to replace chloroquine [9], including artemisinin-based combination therapies (ACTs), which have been recommended by the WHO as the first-line treatment of *P. falciparum* malaria since the 2000s. The group of ACTs include dihydroartemisinin (also named artenimol)–piperaquine, artemether–lumefantrine, artesunate–amodiaquine, artesunate–mefloquine, artesunate–sulfadoxine–pyrimethamine, and artesunate–pyronaridine [10]. Of note, artemisinin clinical resistance was first described in Cambodia in 2008 [11] and is currently emerging in Africa [12,13,14].

We provide an overview on the use of dihydroartemisinin–piperaquine in malaria treatment and discuss tools available for the surveillance of its efficacy.

## 2. Dihydroartemisinin and Piperaquine Mechanisms of Action

**Figure 1:**
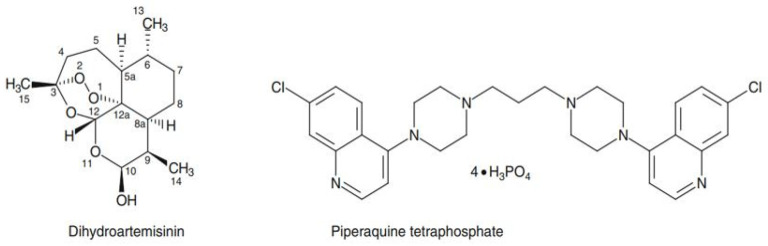
Dihydroartemisinin and piperaquine structure.

Artemisinin was isolated in 1971 from the Artemisia annua plant. This plant was used in traditional Chinese medicine for more than 2000 years to treat fever and malaria [11]. These derivatives kill all stages of asexual parasites, and more particularly the rings (i.e., the youngest form in the intraerythrocytic developmental cycle) and the gametocytes (i.e., the parasites that are infective for the *Anopheles* vectors), reducing the risk of resistance emergence and the mosquitos’ transmission from humans [12,15]. Artemisinin is a sesquiterpene lactone with the empirical formula C_15_H_22_O_5_, and dihydroartemisinin was synthetized from artemisinin in 1973 (Figure 1). The molecule interferes with mitochondrial electrons transportation and heme detoxification in the vacuole [16]. According to Posner et al., endoperoxide bridges of artemisinin derivatives undergo cleavage by reduced heme iron (Fe^2+^ heme), which results in the production of free-radical species that are toxic for the parasite [17]. It induces oxidative stress, proteasomal stress, DNA damage, and alkylation and oxidation of proteins, lipids, and heme [18]. According to Chen et al., dihydroartemisinin can cause morphological variation in the infected erythrocyte membrane, and their work also showed that dihydroartemisinin treatment downregulated the var gene encoding for *P. falciparum* erythrocyte membrane protein 1 (*Pf*EMP1). In addition, *Pf*EMP1 knockout significantly increased sensitivity to artemisinin [19].

**Figure 2:**
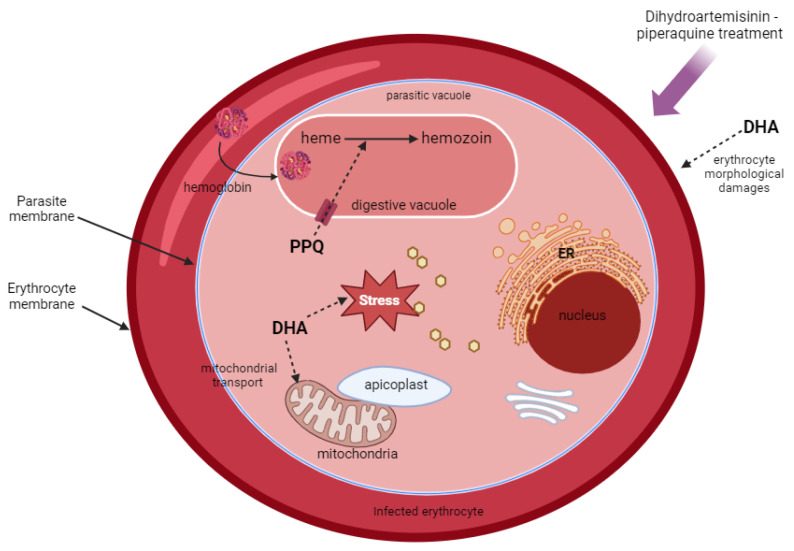
Targets and mechanisms of dihydroartemisinin (DHA) and piperaquine (PPQ) molecules in the parasite *Plasmodium*. ER: endoplasmic reticulum. (Created with BioRender.com).

The piperaquine is a bisquinoline (4-aminoquinolines) that was first synthesized by Rhone-Poulenc in France in the 1960s and was used extensively in Indonesia and China as treatment before becoming a partner molecule in the artemisinin-based combinations (Figure 1) [20,21]. The molecule inhibits heme detoxification and hemoglobin digestion in the digestive vacuole of the parasite which is a similar action as chloroquine [16] (Figure 2).

## 3. Molecular Markers of Resistance to Dihydroartemisinin and Piperaquine

Resistance to artemisinin was first reported in Cambodian clinical studies in 2008, but according to the WHO, it probably preceded the introduction of ACTs in this country [22,23]. Resistances were also reported in Vietnam, Laos, China, and India [23,24,25]. Resistance events have been observed in Uganda and Rwanda, but artemisinin resistance is not yet generalized in Africa [12,13]. The mechanisms of resistance towards artemisinin compounds affect only the ring stage of the parasite, resulting in a delayed parasite clearance which is called “partial resistance” to highlight this time-limited and cycle-specific feature [11]. A parasite clearance half-life above 5 h is associated with “partial resistance” [26,27]. So far, “full” artemisinin resistance has never been reported. In 2014, a single nucleic polymorphism in the gene *PfK13* was identified as molecular marker of artemisinin resistance [28]. *PfK13* is located on the chromosome 13 and encodes a protein essential for asexual erythrocytic development, but its exact function remains unclear. In vitro and in vivo studies have shown that mutations in the PfK13 BTB/POZ and propeller domain are associated with a delayed parasite clearance. To date, more than 260 non-synonymous *PfK13* mutations have been reported, but only 13 have been formally associated with artemisinin partial resistance according to WHO criteria [29] (Table 1). These mutations are associated with parasite clearance half-lives [27,28,30] and increased parasite survival rates according to in vitro ring-stage assays (RSAs) [28,31,32].

However, the main *PfK13* isolates known as associated to artemisinin resistance in Asia, like R539T, Y493H, and I543T, are not reported in Africa [40,41,42,43]. Only three isolates harboring C580Y *PfK13* were detected in Ghana in 2017–2018 [44]. But *PfK13* mutations, less frequent in Asia and now validated for artemisinin resistance, have been identified in Africa, and more particularly in East Africa: in Rwanda with the presence of the A675V, R561H, and P574L mutations and C469F from 2019 [12,45,46,47,48,49,50,51,52], in Uganda with the presence of the A675V and C469Y mutations from 2016 and R561H from 2021 [13,53,54,55,56,57,58], in Tanzania with the presence of the R561H mutation from 2019 and the C469F and A675V mutations from 2021 [59,60,61,62], in Kenya with the presence of the R561H mutation from 2021 and A675V from 2018–2022 [14,63], in Ethiopia with the presence of the R622I new mutation from 2013 [14,64,65], and in Eritrea with the presence of the R622I mutation like in Ethiopia from 2016 and the R561H mutation from 2019 [66]. All these mutations were found in very low proportion except for the R561H C469F and A675V mutations in Uganda (up to 23%, 40%, and 30%, respectively) [13]. The R561H, A675V, and C469F mutations are associated with increased in vitro parasite survival rates [12,48,56] and with parasite clearance half-lives or delayed parasite clearance [50,57]. The A675V mutant was detected in a case of imported malaria from Uganda to France that showed delayed parasite clearance under artesunate treatment [67]. The R622I mutation conferred partial in vitro resistance when edited into an African strain (NF54) [66].

In West Africa, a new mutation C580R/V has been detected in Ghana [68,69]. The R561H mutation was also detected in the Democratic Republic of Congo in 2020–2021 [70].

In 2018, Demas et al. reported the potential implication of the coronin gene in dihydroartemisinin resistance. Three mutations (G50E, R100K, and E107V) were identified in two cultures adapted from the Senegalese *P. falciparum* isolates that became resistant under dihydroartemisinin pressure and were validated via CRISPR-cas9 mutagenesis, after selection drug pressure on Senegalese isolates [33]. This actin-binding protein, located on parasite membranes (parasite plasma, digestive vacuole, and pre-digestive vacuole membranes) is involved in sporozoite mobility [71,72,73]. Moreover, *Pfcoronin* R100K and A107V mutations altered endocytosis and decreased hemoglobin uptake to mediate artemisinin resistance in rings [74]. However, these three mutations have not been found since then, while another mutation, P76S, has been identified in Africa [34,75], and more particularly in Senegal [76], Gabon [35], Ghana [35,77], Kenya [35], Congo [35], Nigeria [78], and Uganda [56]. This mutation has also been reported in cases of artemisinin treatment failures without mutations in *PfK13* [79,80,81]. However, current data do not allow this mutation to be identified as a molecular marker of artemisinin resistance in Africa and no phenotypic association has been yet established with *Pfcoronin* mutations in field isolates [34]. However, isolates with the *PfK13* C469Y mutation associated with the *Pfcoronin* P76S mutation showed greater increases in parasite survival rates (RSAs) than isolates harboring only the *PfK13* C469Y mutation in Uganda [56].

Membrane transporters located in the digestive vacuole membrane are highly involved in antimalarial drug resistance by allowing the efflux of therapeutic molecules from the vacuole [82]. Recently, the *P. falciparum* chloroquine resistance transporter (*Pf*CRT) has been linked with piperaquine resistance. Initially described in chloroquine resistance [83,84], recent studies have demonstrated, more specifically via CRISPR-cas9 mutagenesis, the role of several new mutations (T93S, H97Y, C101F, F145I, I218F, M343L, C350R, and G353V) in the *Pfcrt* gene in piperaquine resistance [37,85,86,87,88]. The T93S, H97Y/L F145I, and I218F mutations were identified in Asian isolates and showed in vitro reduced susceptibility to piperaquine [89,90,91,92]. The C350R mutation, only detected in South American isolates from French Guiana [38,39,93], Suriname [38], and Guyana [38,94], were associated with in vitro decreased piperaquine susceptibility and with dihydroartemisinin–piperaquine treatment failure. However, none of these mutations were detected in African isolates [95]. The involvement of the protein *Pf*CRT in piperaquine resistance is consistent regarding the similar target of chloroquine and piperaquine in the parasite. The F145I mutation leads to lower levels of intracellular total heme in parasites and higher levels of undigested hemoglobin [96].

Moreover, some studies have shown that piperaquine resistance is mediated by amplification of the *plasmepsine II* and *III* (*pm2/3*) genes [36,97,98]. These genes code for two aspartic proteases acting in the hemoglobin degradation in the digestive vacuole [99]. The *pm2/3* amplification was first detected in Asian isolates [98,100,101,102,103]. *Pm2/3* amplification is associated with high rates in in vitro piperaquine survival assays (PSAs) and with dihydroartemisinin–piperaquine treatment failures [36,104,105]. The proportion of parasites with multiple copies of *pm2/3* can be high in Asian areas, like in Vietnam in 2018–2019 with a rate of 56% [103]. Nucleic amplification in these genetic regions has also been found in Guyana isolates harboring a high survival rate after in vitro contact with a high concentration of piperaquine [38]. The occurrence of *pm2/3* amplification and *Pfcrt* C350R was described in 15% of the isolates from Guyana in 2021 [94]. There is evidence that *pm2/3* amplification occurred in Africa between 2016 and 2021 [106,107,108], but the proportions of isolates with multiple copies of *pm2/3* are mostly very low in Africa like in Senegal [109], Uganda [54,110,111], Sudan [112], Mozambique [113,114], Kenya [115], Angola [116], Tanzania [117], Sierra Leone [118], and Mali [119]. However, its association with piperaquine in vitro reduced susceptibility or high PSA survival rates, or with dihydroartemisinin–piperaquine treatment failures, was not confirmed in African isolates [106,120]. Each additional copy of *pm2/3* increases PSA survival rate in Dd2 clones harboring the M343L mutation in the *Pfcrt* gene [121].

## 4. Dihydroartemisinine and Piperaquine Phenotypic Susceptibility Testing

Parasites’ susceptibility profiles towards molecules are studied using the ring-stage survival assay for artemisinin and the piperaquine survival assay (RSA-PSA), providing a survival rate of parasites after exposure to a specific molecule concentration [31,104], or through dose-response chemosusceptibility tests quantifying parasites after exposure to a panel of drug concentrations to determine the half maximal inhibitory concentration (IC_50_) which is the molecule rate that inhibits the parasites growth by 50% [122].

**Figure 3:**
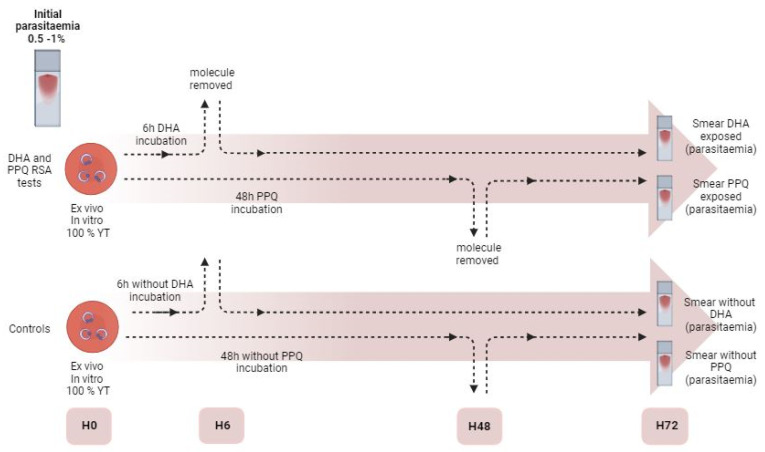
RSA-PSA tests template. The initial parasitemia must be between 0.5 and 1% with 100% of young trophozoite (rings). This figure was developed from a template document generated by the WorldWide Antimalarial Resistance Network (WWARN). The original document is available on the WWARN website www.wwarn.org (accessed on 9 November 2024) (Created with BioRender.com).

The RSA-PSA tests were specifically developed to identified artemisinin and piperaquine resistance. They can be performed on ex vivo isolates as well as on in vitro cultures, using a percoll gradient to only select the ring stage to be studied. Young trophozoite parasites are exposed to 200 nM piperaquine for 48 h or 700 nM artemisinin for 6 h. A growth rate is calculated from the initial smear parasitemia and the smear parasitemia after 72 h. According to Witowski et al., the growth rate in negative controls (unexposed to the molecule) must be greater than 1 for ex vivo parasites and greater than 1.5 for in vitro parasites for quality control of the experimentation [31]. The survival rate of parasites to artemisinin or piperaquine is calculated by the ratio calculated by dividing the parasitemia at 72 h among parasites exposed to the drug by the parasitemia at 72 h among controls (unexposed parasites) (Figure 3).

**Figure 4:**
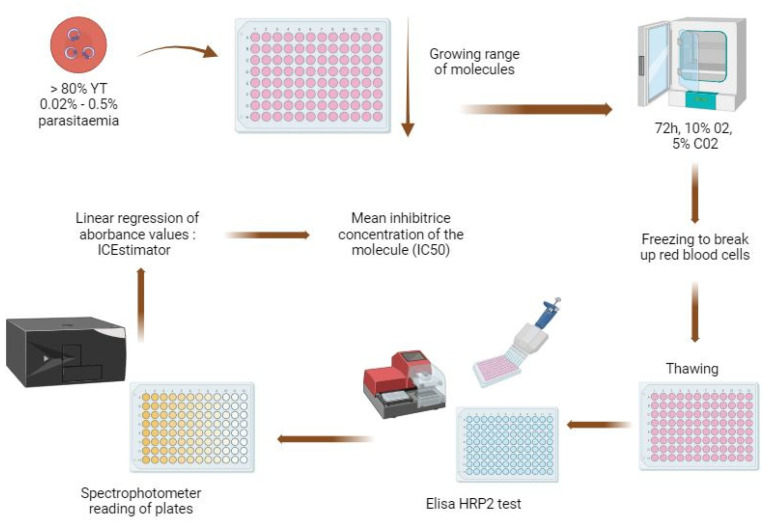
Chemosusceptibility test template. The test should be carried out with parasitemia adjusted to 0.05% and a minimum of 80% young trophozoites. Incubation with drugs lasts 72 h in a controlled atmosphere at 37 °C with 10% O_2_ and 5% CO_2_. After 72 h of incubation, the plates are frozen to allow lysis of the red blood cells, HRP2-based ELISA assays are used to measure the parasites’ growth, and the 50% inhibitory concentration (IC_50_) is determined via non-linear regression. (Created with BioRender.com).

To perform standard antimalarial chemosusceptibility tests, parasites with parasitemia adjusted to 0.05% with a minimum of 80% young trophozoites are incubated with an increasing range of molecule concentrations for 72 h. After drug exposure, parasites are quantified by using colorimetric enzyme-linked immunosorbent assays (ELISAs) that recognize the specific *P. falciparum* histidine-rich protein (PfHRP2) [123] or by using fluorescent nucleic acid intercalating dyes, such as SYBER green I, that only detect malarial DNA since mature erythrocytes lack RNA and DNA (Figure 4) [124]. Isotopic tests with tritiated hypoxanthine adapted from Desjardins can also be used to determine inhibitory concentrations at 48 h [125,126].

Chemosusceptibility tests can be routinely performed ex vivo on clinical isolates for several therapeutic molecules, getting results within a few days after the samples’ collection. Then, IC_50_ values are estimated via non-linear regression and compared to the resistance thresholds that have been estimated at 10.5 nM for dihydroartemisinin and 135 nM for piperaquine using the same laboratory protocol as applied to clinical samples [127,128].

## 5. Dihydroartemisinin and Piperaquine Clinical Use, Safety, and Therapeutic Efficacy

Dihydroartemisinin is rapidly absorbed, reaching a maximum plasmatic concentration (Tmax) in 1–2 h and has an elimination half-life of around one hour. The piperaquine is slowly absorbed with a Tmax around 5 h and an elimination half-life of around 22 days in adults and 20 days in children [129]. Clinical studies of this combination did not recruit patients aged over 65 years nor pregnant women. However, studies showed similar pharmacokinetic profiles in pregnant and non-pregnant women, and based on reports, its use appeared safe during pregnancy [130,131,132].

Piperaquine is metabolized by the cytochrome CYP3A4 in the liver, so that CYP3A4 enzyme-inducing drugs such as rifampin, carbamazepine, phenytoin, phenobarbital, and St. John’s wort (*Hypericum perforatum*) are likely to reduce piperaquine plasmatic rates [129].

Piperaquine may have cardiac side effects by inducing QT interval prolongation, tachycardia, or bradycardia due to delayed ventricular depolarization. An observational study carried out in Burkina Faso, Mozambique, Ghana, and Tanzania showed that the QTc interval could exceed 500 ms (3 per 1002) after the third dose, and that an interval greater than 60 ms was detected in less than 10% of patients in the study with no clinical abnormalities [133,134]. European studies reported a good clinical tolerance [135] without significant increase in the risk of unexplained sudden death than in the general population [136]. Thus, piperaquine must be administered carefully in patients with prolonged QT or receiving other medications inducing QT interval prolongation.

In the 2000s, public–private partnerships between Medicines for Malaria Venture, Sigma-Tau Industrie Farmaceutiche Riunite SpA, and other academic and industrial partners across the world led to the development of a dihydroartemisinin–piperaquine formulation (Eurartesim^®^) manufactured according to international Good Manufacturing Practice standards. Eurartesim efficacy and safety was investigated in two large phase III randomized, open-label clinical trials in patients with uncomplicated *P. falciparum* malaria. In the first study, Eurartesim was compared with another malaria medicine containing artesunate and mefloquine in 1150 Asian adult and pediatric patients. The main measure of effectiveness was the proportion of patients who were cured 63 days after treatment. In the second study, Eurartesim was compared with another medicine containing artemether and lumefantrine in 1553 African children. The main measure of effectiveness was the proportion of patients who were cured 28 days after treatment. In each case, the results confirmed that Eurartesim was not inferior to the comparator medicinal product. In both studies, the true treatment failure rate was below the 5% efficacy threshold set by the WHO and led to the development of Eurartesim to international regulatory standards [137]. In 2011, Eurartesim received marketing authorization in Europe via a centralized procedure by the European Medicine Agency (EMA) for uncomplicated episodes of *P. falciparum* malaria as film-coated tablets containing 160 mg piperaquine with 20 mg dihydroartemisinin and 320 mg piperaquine with 40 mg dihydroartemisinin. The drug, licensed for its use in children above 6 months of age and adults, must be administrated according to the recommended dosing listed in the summary of product characteristics last updated in 2023 presented in Table 2a, in the absence of vomiting, and as a relay to the initial intravenous artesunate treatment in severe malaria cases. It is administered once a day for three days, on an empty stomach, fasting at least 3 h before and after the drug ingestion. The reason is that piperaquine absorption is increased in the presence of fatty foods, which may increase its effect on the QTc interval. The dose is adapted according to the patient’s body weight, but no data are available for patients weighing more than 100 kilos [129]. Other weight-based regimens have been used in clinical trials (Supplementary Table S1) and may be recommended by manufacturers for other marketing dihydroartemisinin–piperaquine formulations, as for example Duo-Cotecxin^®^ [138], or may be proposed as alternative standards such as in Médecin Sans Frontières (MSF) medical guidelines [139]. Reported daily doses may range from 1.6 to 4 mg/kg of dihydroartemisinin and from 12.8 to 32 mg/kg of piperaquine. But importantly, Eurartesim was reported with a better stability in comparison with other DHA-based formulations [140]. For comparison purposes, the doses received for a given weight under three different regimens are presented in Table 2b.

The WHO recommends regular surveillance of antimalarial efficacy notably in endemic areas according to a standard protocol of efficacy assessment and follow-up [141]. The main endpoint in therapeutic efficacy studies (TESs) generally corresponds to the adequate clinical and parasitological response at day 42 after the initial dose of dihydroartemisinin–piperaquine as it is performed in real life. Some intermediate outcome measures based on physical and microscopic blood examination are recommended by the WHO during TESs on days 1, 2, 3, 7, 14, 21, 28, and 35 [142]. To monitor the performance of dihydroartemisinin–piperaquine TESs have been conducted in various indications in recent years and are referenced in ClinicalTrials.gov (Supplementary Table S1). Most of the TESs in endemic areas, both in children and adults with uncomplicated *P. falciparum* malaria, found that a dihydroartemisinin–piperaquine combination remained efficacious per the WHO’s 90% cutoff in the confidence interval and was well tolerated [138,143,144,145,146,147]. Likewise in pregnant women recruited in areas with widespread artemisinin resistance and in Africa, dihydroartemisinin–piperaquine provided satisfactory efficacy [132,148]. Recent metanalyses on TESs conducted in sub-Saharan Africa confirmed the dihydroartemisinin–piperaquine malaria treatment’s success above the WHO threshold value (91% and 99% after PCR correction) [149,150]. In Vietnam, the efficacy of dihydroartemisinin–piperaquine is still satisfactory, but the delayed parasite clearance time and the high positivity rate at day 3 of treatment are indicative of emerging artemisinin resistance and close monitoring of dihydroartemisinin–piperaquine efficacy in this area is advocated [101,151]. However, across the eastern Greater Mekong subregion (northeastern Thailand, western Cambodia, northeastern Cambodia, and southwestern Vietnam), dihydroartemisinin–piperaquine is no longer treating malaria effectively, reaching 50% efficacy at day 42 (95% CI 41.1–58.3) in a 2015–2018 trial which has highlighted a major increase in parasites harboring molecular markers of artemisinin resistance (*kelch13* Cys580Tyr mutations) and piperaquine resistance (plasmepsin2/3 amplification and four mutations in the *Pfcrt* gene (Thr93Ser, His97Tyr, Phe145Ile, and Ile218Phe) [152], as confirmed in an observational study in Cambodia [30].

Performance of combinations associating dihydroartemisinin–piperaquine with other molecules such as imatimib or mefloquine are being evaluated and could be promising, notably in areas with multi-drug resistances [152] (Supplementary Table S1). 

Moreover, dihydroartemisinin–piperaquine offers an interesting post-treatment prophylactic effect following therapy, reducing the risk of new infection, an issue of relevance in highly endemic malaria countries. However, the real impact on gametocytes carriage and malaria transmission to mosquitos remains uncertain [153]. In that aim, association with low-dose primaquine may be helpful [154,155] and well tolerated [156], as well as an association with methylene blue which is an interesting gametocytocidal compound [157].

In addition, several TESs have provided evidence of a high cure rate in patients with *P. vivax* malaria treated with dihydroartemisinin–piperaquine which could have an improved post-treatment prophylactic effect lasting for up to six weeks [158].

Moreover, dihydroartemisinin–piperaquine has been successfully evaluated for chemoprevention as an intermittent preventive treatment in pregnancy [159,160,161,162], in school-aged children [163,164,165], or in other specific populations and also during mass treatment strategies alone or in association with other drugs to target malaria elimination (Supplementary Table S1). But its preventive efficacy appears dependent on the dosing strategy and rate of piperaquine exposure [166] which raises major concerns about the risk of resistance emergence. All the more important that preventive strategies based on piperaquine must carefully determine the correct dosing regarding the potential cardiac side effects as one previous study evaluating the safety and efficacy of a monthly 2-day regimen using daily doses of 180 mg dihydroartemisinin and 1440 mg piperaquine in healthy military volunteers deployed along the Thai–Cambodian border revealed a mean QTcF prolongation of 46 ms over placebo at the maximum concentration of drug in serum on day 2 [167]. A strategy of monthly malaria prophylaxis with a 3-day course of weight-based dihydroartemisinin–piperaquine associated with 22.5 mg weekly primaquine for 12 weeks is being compared with focused screening and treating in Cambodian military forces [168].

**Figure 5:**
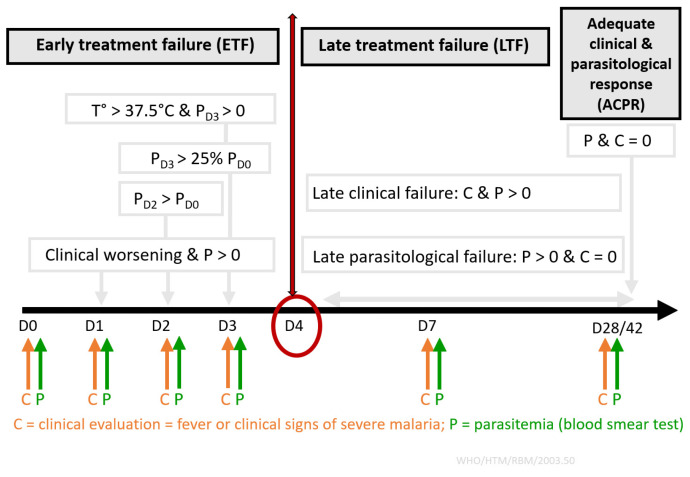
WHO definitions for antimalarial drug responses.

For standard clinical management, malaria treatment efficacy in patient is monitored via clinical examination and a blood smear test after the start of the treatment at day 3, 7, and 42 [169], noting that the endpoint is day 28 for other ACTs which is due to the longer half-life of piperaquine [141]. According to the WHO guidelines, there are four types of drug responses (Figure 5) [141].

Early therapeutic failure (ETF) is defined either as the diagnosis of severe malaria between day 1 and day 3 or by the presence of fever on day 3 after treatment with parasitemia higher than 0 or by parasitemia at day 2 over parasitemia at day 0 or by parasitemia at day 3 higher than 25% of parasitemia at day 0. Parasitological late failure (PLF) is defined as the detection of parasites in the blood without clinical signs between day 4 and day 28/42. Late clinical failure (LCF) is defined as the diagnosis of severe malaria or the presence of fever associated with positive parasitemia between day 4 and day 28/42. Finally, an adequate response is defined as complete parasitic clearance at day 28/42 without criteria for ETF, PLF, or LCF, suggesting parasites’ complete susceptibility to the treatment. It has even been shown that restricting the follow-up of clinical surveillance or clinical efficacy trials to day 42 for dihydroartemisinin–piperaquine could miss a proportion of LCF [170].

## 6. Dihydroartemisinin and Piperaquine Therapeutic Failures

First of all, therapeutic failures as defined by the WHO are not necessarily due to parasites’ resistance to the treatment but may be due to other factors such as under exposition to the drug or reinfection in endemic areas. For example, therapeutic failures have been linked to dihydroartemisinin–piperaquine underdosage in obese patients [171,172]. That is why any therapeutic failure should be investigated starting with the confirmation of the correct drug administration and metabolism, ideally confirmed via plasmatic dosages. Plasma venous piperaquine concentrations below 30 ng/mL on day 7 are predictive of treatment failures [173,174,175] while the cut-off for capillary blood is below 57 ng/mL [174,176,177]. Predicted venous or capillary plasma concentrations are below 10 ng/mL at day 28 [178,179]. Moreover, ACT therapeutic failures must be more particularly monitored in non-endemic areas where there is no possibility of new infection and given the greater probability of non-immune travelers to develop symptomatic recrudescence [180].

Artemisinin partial resistance alone rarely leads to clinical treatment failure, but the emergence of resistance to the partner drugs is of major concern for ACTs’ efficacy. Importantly, the association dihydroartemisinin–piperaquine demonstrated an overall efficacy rate above 99% in areas free of resistance [181]. The clinical efficacy of ACTs is monitored by the WHO through therapeutic efficacy studies conducted at regular intervals at the same sites to detect declines in drug efficacy early and to guide local policies. Piperaquine treatment failures were observed during a clinical trial conduced in 1972–1974 in the country of Hainan Island with recrudescence of infection occurring 20 days after this monotherapy, and major piperaquine therapeutic failures were further reported in the same region before the introduction of ACTs with piperaquine as a partner drug [182,183,184]. Dihydroartemisinin–piperaquine has been recommended since 2008 by the WHO as the first-line treatment for uncomplicated *P. falciparum* malaria in Cambodia, where artemisinin resistances have promptly emerged and spread in the Mekong region since then. The association of dihydroartemisinin and piperaquine appears to remain highly effective to treat *P. falciparum* infection in Indonesia and is rather effective in China and Myanmar [185,186]. However, according to Van der Pluijm et al., this combination is no longer efficient enough in other countries of the Greater Mekong subregion, where *Pfk13*, *Pfcrt* mutations, and *pm2/3* amplification are highly prevalent [187].

Based on molecules’ elimination half-lives, early failure is suspected to occur because of artemisinin partial resistance, whereas resistance to the partner drug piperaquine is highly suspected in late failure [129]. However, we reported a case of severe imported *P. falciparum* malaria with parasitemia remaining positive at day 7 under artesunate treatment, without polymorphism in the *PfK13* gene, highlighting the limits of standard definitions for in vivo resistance [188]. Moreover, early or late therapeutic failure have been reported in the absence of genetic polymorphism associated with artemisinin or piperaquine resistance, illustrating the lack of reliable markers for malaria treatment resistance, mainly in Africa (Table 3), and the valuable input of chemosensitivity testing.

## 7. The Need for New Tools for the Surveillance of Dihydroartemisinin–Piperaquine Efficacy

The RSA-PSA tests have become the gold standard for artemisinin and piperaquine susceptibility phenotyping, but they have some limitations. Ex vivo isolates must be fresh and intact; if not, they must be cultured, but isolates may fail to adapt to the culture. Testing on asynchronous cultures is not possible so that several steps of synchronization are required, using sorbitol and a percoll gradient which delay the results. Somes studies had also demonstrated the potential bias of parasite adaptation to culture by selecting parasite subpopulations [194]. Moreover, parasite quantification using smear readings is operator dependent. This may be addressed by repeating the reading with different experienced operators or by using flow cytometry analysis to measure parasitized red blood cells [195].

Chemosusceptibility assessment via HRP2-based ELISA, SYBR green I, or isotopic assays can be used routinely ex vivo on clinical isolates, getting results within a few days after the samples’ collection. However, it may fail with low parasitemia, and it lacks standardization between labs often making its results not comparable. Moreover, difficulties have been reported in the interpretation of piperaquine assay results. In vitro dose–response curves are frequently yielded non-interpretable and laboratory dependent [37,104]. Moreover, some therapeutic failures remain unexplained by the values of piperaquine IC_50_ [196]. Thus, phenotyping tests are used to monitor clinical isolates’ sensitivity to antimalarial drugs but have no impact on the choice of treatment [9].

Likewise, reliable molecular markers of drug resistance, i.e., highly sensitive, specific, and universal, are lacking. For example, unlike in South America, piperaquine resistance does not appear to be linked with plasmepsin genes’ amplification in Cambodia, China, or Maynar [38,93,186,197], and studies have demonstrated that the amplification of the plasmepsin II gene did not lead to reduced piperaquine susceptibility in African isolates [106,120,198]. Concerning artemisinin, to date, *PfK13* mutations, and more specifically new mutations, have been identified in East Africa [199,200]. These mutations are found in very low proportions, with a percentage that does not reach the resistance threshold set by the WHO except for the R561H C469F and A675V mutations in Uganda (up to 23%, 40%, and 30%, respectively) [13]. Indeed, it has been shown that genetic analysis indicated the local emergence of mutant parasites independent of those previously reported in Southeast Asia [12,201]. The list of validated and candidate markers is continually being updated by the WHO based on criteria for correlation between mutations and clearance phenotypes. However, this correlation rate is far from 100%, and polymorphisms seem to have major geographic specificities.

## 8. Conclusions

Dihydroartemisinin–piperaquine combination remains a corner stone in the treatment of uncomplicated malaria infections. The emergence of resistance towards the two molecules threatens its effectiveness. The lack of reliable markers and tests to assess partial or full resistance make the surveillance of treatment efficacy necessarily multidimensional, relying on clinical studies, records of therapeutic failures, routine strain phenotyping using several drug susceptibility assays, and the sequencing of validated and candidate genes potentially involved in resistance mechanisms. The whole genome analysis of isolates from patients with confirmed therapeutic failure will be helpful to identify new genome variations involved in resistance. Whole genome sequencing could also enable a more in-depth investigation of polyclonal infections with the bio-informatics pipeline. The next step will probably be the large-scale implementation of the whole genome molecular surveillance of parasites [202]. Lastly, the malaria therapeutic arsenal needs to be developed, including new compounds, resistance reversers, or new strategies such as drug repurposing or the use of triple- or quadri-therapy to reduce resistance emergence.

## Figures and Tables

**Table 1:** Candidate and validated molecular markers of dihydroartemisinin and piperaquine resistance, as of august 2024.

**Molecule**	**Gene**	
**Dihydroartemisinin**	*PfK13*	F446I, N458Y, C469Y, M476I, Y493H, R539T, I543T, P553L, R561H, P574L, C580Y, R622I, A675V [29]
	*PfK13* Candidate	P441L, G449A, C469F, A481V, R515K, P527H, N537I/D, G538V, V568G [29]
	*Pfcoronin* *	G50E, R100K, E107V [33]
		P76S [34,35]
**Piperaquine**	*PfPlasmepsin II/III*	Number of copy > 1 [36]
	*Pfcrt*	T93S, H97Y, F145I, M343L, G353V [37] C350R [38,39]

* to be further confirmed.

**Table 2:** (**a**) Daily dose of dihydroartemisinin–piperaquine according to the patient body weight and the marketing authorization by the European Union (EMA summary of product characteristics). (**b**) Daily therapeutic doses of dihydroartemisinin and piperaquine in mg (dihydroartemisinin/piperaquine) administrated for a given weight under 3 different regimens.

**(a)**			
**Weight** **(kg)**	**Daily Dose (mg)**		**Tablet Strength and Number of** **Tablets per Dose**
	**Piperaquine**	**Dihydroartemisinin** **(artenimol)**	
5 to <7	80	10	½ tablet (160/20 mg)
7 to <13	160	20	1 tablet (160/20 mg)
13 to <24	320	40	1 tablet (320/40 mg)
24 to <36	640	80	2 tablets (320/40 mg)
36 to <75	960	120	3 tablets (320/40 mg)
>75	1280	160	4 tablets (320/40 mg)
(**b**)			
**Body Weight (kg)**	**Regimen 1** **Eurartesim EMA Recommendations**	**Regimen 2** **MSF Recommendations**	**Regimen 3** **Weight-Based 2.4 mg/kg Dihydroartemisinin and 20 mg/kg Piperaquine**
5	10/80	20/160	12/100
10	20/160	25/240	24/200
15	40/320	40/320	36/300
20	40/320	60/480	48/400
25	80/640	80/640	60/500
30	80/640	80/640	72/600
40	120/960	120/960	96/800
50	120/960	120/960	120/1000
60	120/960	160/1280	144/1200
70	120/960	160/1280	168/1400
80	160/1280	200/1600	192/1600
90	160/1280	200/1600	216/1800
100	160/1280	200/1600	240/2000

**Table 3:** Dihydroartemisinin–piperaquine therapeutic failures unexplained by molecular marker for resistance in Africa (ND: no data, so partially explored).

Reference	Clinical’s Data (Treatment on First Access)	Gene			Countries (Travel Date)
		PfK13	Pfpm2	Other	
Gobbi et al. [189]	Dihydroartemisinin–piperaquine Recurrent episode: 32 days after diagnosis Correct treatment adsorption	WT	ND	*Pfcrt*: M74I, N75E, K76T *Pfmdr1*: D1246Y *Pfdhps*: A437G, K540E *Pfdhfr*: C59R, S108N	Ethiopia (2014)
Malvy et al. [190]	Dihydroartemisinin–piperaquine Supervised intake Recurrent episode: 2 years post treatment at 24 weeks pregnant	WT	1 copy	WT	Cameroon (2014)
Russo et al. [191]	Dihydroartemisinin–piperaquine Recurrent episode: 30 days after diagnosis Supervised intake	WT (C580)	1 copy	*Pfcrt:* M74I, N75E, K76T *Pfmdr1*: Y184F *Pfdhfr*: C59R, S108N *Pfdhps*: K540E	Ethiopia (2016)
Motta et al. [192]	artesunate in IV followed by dihydroartemisinin–piperaquine Recurrent episode: 30 days after diagnosis Supervised intake	WT	1 copy	*Pfdhps*: S436A *Pfdhfr*: C59R, S108N	Ivory Coast/ Abidjan (ND)
Pierreux et al. [80]	Dihydroartemisinin–piperaquine Recurrent episode: 25 days after diagnosis Supervised intake	WT	ND	*Pfcoronin*: S183G *Pfdhfr*: N51I, C59R, S108N	Cameroun (2023)
Delandre et al. [193]	Dihydroartemisinin–piperaquine Recurrent episode: 32 days after diagnosis. Supervised intake and correct treatment adsorption	WT	1 copy	*Pfdhfr*: N51I, C59R, S108N	Comoros (2023)

## Supplementary Materials

The following supporting information can be downloaded at https://www.mdpi.com/article/10.3390/jcm13226828/s1, Supplementary Table S1: List of therapeutic efficacy studies (TES) on dihydroartemisinin piperaquine recorded in ClinicalTrial as of 1 November 2024 (total trials = 142, TES = 50).
